# Isolation and Genetic Characterization of African Swine Fever Virus from Domestic Pig Farms in South Korea, 2019

**DOI:** 10.3390/v12111237

**Published:** 2020-10-30

**Authors:** Hyun-Joo Kim, Ki-Hyun Cho, Ji-Hyoung Ryu, Min-Kyung Jang, Ha-Gyeong Chae, Ji-Da Choi, Jin-Ju Nah, Yong-Joo Kim, Hae-Eun Kang

**Affiliations:** Animal and Plant Quarantine Agency, 177 Hyeoksin 8-ro, Gimcheon 39660, Korea; vetkhj@korea.kr (H.-J.K.); vet10@korea.kr (K.-H.C.); yjh562@naver.com (J.-H.R.); ckone0506@hanmail.net (M.-K.J.); cogkrud@naver.com (H.-G.C.); apjida@korea.kr (J.-D.C.); nahjj75@korea.kr (J.-J.N.); kyjvet@korea.kr (Y.-J.K.)

**Keywords:** African swine fever, virus isolation, genetic characterization, South Korea

## Abstract

On 17 September 2019, the first outbreak of African swine fever in a pig farm was confirmed in South Korea. By 9 October, 14 outbreaks of ASF in domestic pigs had been diagnosed in 4 cities/counties. We isolated viruses from all infected farms and performed genetic characterization. The phylogenetic analysis showed that all of fourteen ASFV isolates in South Korea belong to genotype II and serogroup 8. Additionally, all isolates had an intergenic region (IGR) II variant with additional tandem repeat sequences (TRSs) between the I73R and I329L genes and showed characteristics of central variable region (CVR) 1 of the B602L gene and IGR 1 of MGF 505 9R/10R genes. These are identical to the genetic characteristics of some European isolates and Chinese isolates.

## 1. Introduction

African swine fever (ASF) is a fatal viral disease that affects domestic and wild pigs of all ages around the world. ASF virus (ASFV) is highly virulent and remains a global threat because no effective vaccines are available to prevent the disease. Since ASF was introduced to Georgia from Africa in 2007, it has spread to neighboring countries including Russia, and EU member countries in 2014. In Asia, ASF was first reported in China in August 2018 [1] and spread to Mongolia, Vietnam, Cambodia, North and South Korea, Laos, the Philippines, Myanmar, Timor L’este, Indonesia in 2019, and India in 2020.

The first ASF case in South Korea was reported on 16 September 2019 at a pig farm located in Paju city, Gyeonggi-do province [2]. Over 24 days, from 16 September to 9 October, 14 cases of ASF were confirmed in 4 cities/counties (5 cases in Paju city, 2 in Yeoncheon county, 2 in Gimpo city, 5 in Ganghwa county) following along the borderline between South and North Korea. On 3 October 2019, the first carcass of a wild boar infected by ASFV was found in the demilitarized zone (DMZ), at a distance of 8.4 km from the 14th ASF outbreak pig farm in Yeoncheon county [3]. Since then, up to 754 cases of ASFV-infected wild boars had steadily been found or captured in Paju, Yeoncheon, Pocheon, Cheorwon, Hawcheon, Yanggu, Goseong, Inje, and Chuncheon by 30 September 2020.

In previous studies, we reported that the first ASFV isolate Korea/Pig/Paju1/2019 belongs to genotype II and has an additional tandem repeat sequence in the intergenic region (IGR) between the I73R and I329R gene, and the same characteristics were reported in the wild boar [2,3]. In this study, we isolated ASFVs from all 14 infected domestic pig farms and analyzed molecular characteristics of all isolates to find any differences between them, and to compare with characteristics of ASFVs isolated in Europe and Asia. As there is little genetic difference in the same genotype based on the B646L gene of ASFV, investigations for less-conserved regions have been conducted to identify genetic differences between isolates within the same genotype. As a result of these studies, the classification of the serogroup-based EP402R gene, different types of central variable region (CVR) of the B602L gene, and kinds of tandem repeat sequence (TRS) insertion in the IGR of multigene family (MGF) 505 9R/10R genes were reported [4,5,6,7]. CVRs can be used for the intra-genotypic differentiation of isolates within the same genotype, and TRS insertion in the IGR of MGF 505 9R/10R is a new marker of genetic variation among ASFV of genotype II.

For appropriate and adequate control measures against ASFV, the genetic characterization of isolates is required. We isolated ASFV from 14 infected domestic pig farms and identified both the characterization of isolates in vitro and additional genetic properties in this study.

## 2. Materials and Methods

### 2.1. Virus Isolation

EDTA-treated whole blood and spleen were collected from infected domestic pig farms. The spleen was homogenized and supernatant after centrifugation was used to inoculate porcine alveolar macrophage (PAM) for virus isolation, in accordance with the procedure established by the Center for Animal Health Research (CISA), the European Union Reference Laboratory of ASF and OIE [8]. In brief, PAM cells were seeded in 96-well tissue culture plates and incubated for 4 h at 37 °C in a CO_2_ incubator. Prepared samples were inoculated and 1% pig erythrocyte suspension added. The virus in the cell supernatants was confirmed by qPCR and virus isolation was confirmed by hemadsorption (HAD).

### 2.2. PCR Assay

Nucleic acids were extracted using Maxwell^®^ RSC Total Nucleic Acid kit and Maxwell RSC Whole Blood DNA extraction kit (Promega, Madison, Wisconsin, USA). For genetic analysis of ASFV, we amplified the B646L gene encoding the p72 for genotype, the EP402R gene encoding the CD2v for serogroup, CVR within the B602L gene, and IGR between the I73R and I329L genes as previously reported [7,9,10,11]. For amplification of the IGR between the MGF 505 9R/10R genes, we designed a set of primers, forward (5′-AGA TTG CAG AAA CCG CAG AT-3′) and reverse (5′-AGC CAA GGG GTA AGG AAG AA-3′), using the PRIMER 3 program (http://primer3.ut.ee). PCR was performed with a protocol as follows: 10 min at 94 °C, followed by 40 cycles of 30 s at 94 °C, 1 min at 55 °C, 1 min at 72 °C, with a final extension of 10 min at 72 °C. PCR products were submitted to Macrogen for sequencing (Daejeon, South Korea).

### 2.3. Analysis of the ASFV Isolates

All sequences were analyzed using BioEdit version 7.2 (Ibis Biosciences, http://www.mbio.ncsu.edu/bioedit/bioedit.html). Phylogenetic analysis of nucleotide sequences of the p72 gene for genotype and the EP402R gene for serogroup were conducted using MEGA 6.0 software (http://www.megasoftware.net).

## 3. Results and Discussion

**Figure 1:**
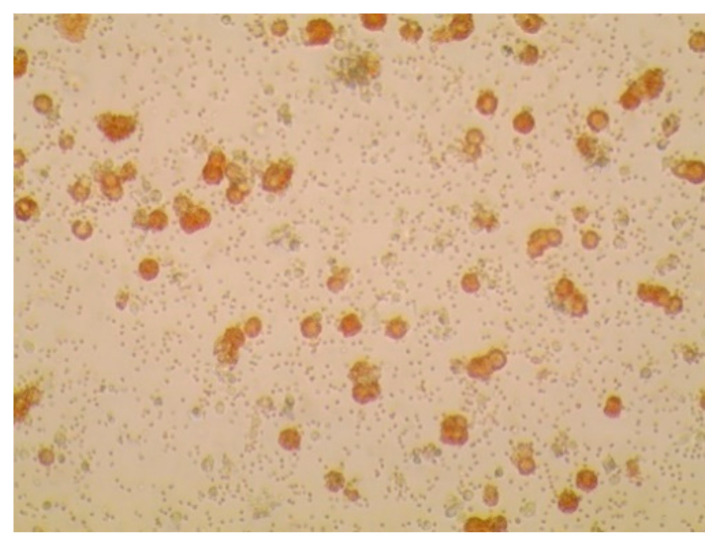
Hemadsorption (HAD) assay for the detection of African swine fever virus (ASFV) in a field sample of spleen tissue. The 10 times dilution of the supernatant of the homogenate was inoculated into porcine alveolar macrophage (PAMs) with 1% pig blood cells. HAD was observed within 24 h. Original magnification 100×.

As shown in Table 1, ASFVs were successfully isolated from the blood or spleen of infected domestic pigs. We isolated the 14 ASFVs using primary porcine alveolar macrophages (PAMs), as described in the manual of OIE, which showed HAD phenomenon (Figure 1) within 24 h. Cycle threshold (*C*_t_) values of 14 ASFV isolates were between 13.3 and 18.1 (Table 1).

For genetic analysis of ASFV isolates, we amplified the B646L gene encoding the p72, EP402R gene encoding the CD2v, CVR within the B602L gene, IGR between the I73R and I329L genes, and IGR between the MGF 505 9R and 10R genes. All sequences of Korea/Pig/Paju1/2019 were deposited into GenBank (accession nos. MN603967, MT335858, MN631140, MN603969, and MN603968).

All of the other 13 isolates were classified genotype II and had the IGR II variant between the I73R and I329L genes, as was previously reported in Korea/Pig/Paju1/2019 (Table 1) [2]. Chinese ASFV isolates were divided into three IGR variants: IGR I variant (in only one case of wild boar) [12], IGR II variant (in most cases of domestic pigs), and IGR III variant with the extra insertion of TRS recently reported by Ge et al. [13]. However, only the IGR II variant was reported from domestic pigs and wild boars in South Korea [2,3]. Most recently, as the outbreak of wild boars continued, IGR I and IGR III variants were reported from wild boars in South Korea [14]. Unlike wild boars, it is assumed that IGR variants did not appear in domestic pigs because the outbreak was stopped in a short period.

**Figure 2:**
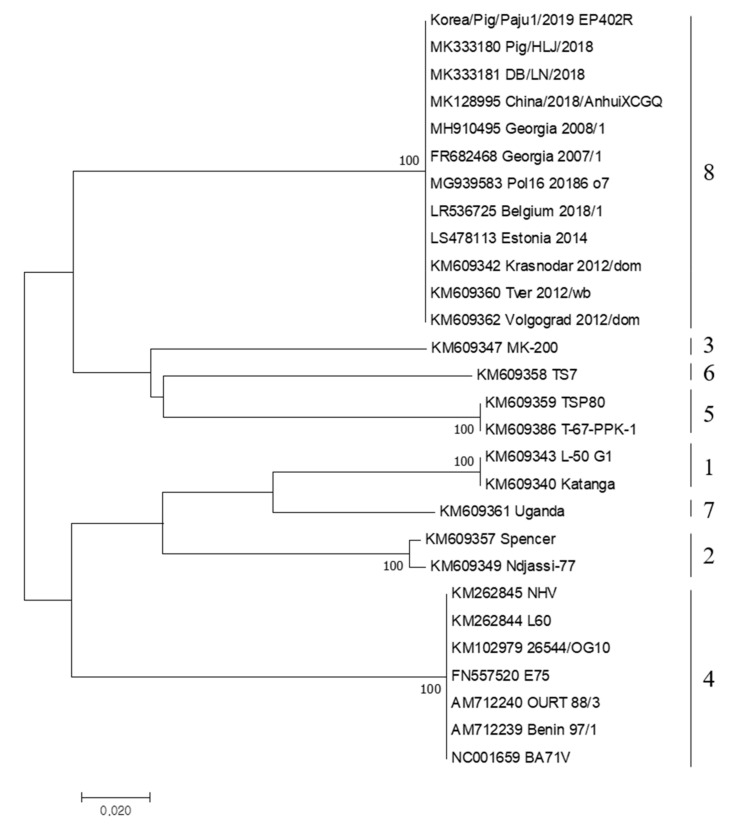
Phylogenetic tree of ASFV isolated in South Korea in 2019 based on partial sequence of CD2v serogroup. Neighbor-joining phylogenic tree was constructed using MEGA6.0 software.

In addition, we compared the EP402R gene sequence for serogroup classification. The EP402R gene encodes the CD2v protein, which is related to the HAD phenomenon [4] and is proposed as a new genetic marker for the classification of ASFV other than genotype [10]. The results of serotyping based on the EP402R gene were all serogroup 8 (Figure 2). China/2018/1 and Korean wild boar isolate 19S804 were also reported as serogroup 8 [3,13], and almost all ASFVs genotype II isolated recently in Europe and Asia were confirmed to belong to serogroup 8.

**Figure 3:**
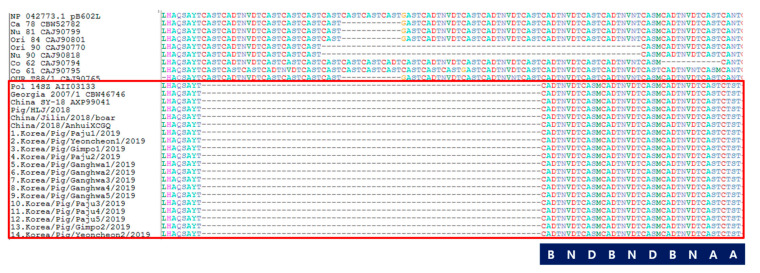
Central hypervariable region (CVR) analysis of ASFV isolated in domestic pigs of South Korea, 2019. Code as labeled in previous studies; [6]. A = CAST, CVST, CTST, or CASI; B = CADT, CADI, CTDT, or CAGT; D = CASM; N = NVDT, NVGT, NVDI, or NCDT.

We also analyzed the CVR profile of the B602L gene, and the IGR of MGF 505 9R/10R, as new markers to differentiate isolates in the same genotype of ASFV as described in [5,6,7]. The CVR of the B602L gene could show distinct variability in the nucleotide sequence, and it was translated into the amino acid sequence and grouped by methods reported in [6]. Amino acid tetramer codes are provided in Figure 3. It could be one of the most useful regions for tracing the source of the outbreak. Only one type of tandem amino acid repeat sequence profile within the CVR (BNDBNDBNAA) was identified among Korean isolates. This CVR profile was 100% identical to that of Georgia 2007/1, Pol14/Sz, China SY-18, China/Jilin/2018, China/Pig/HLJ/2018, and China/2018/AnhuiXCGQ, even though there are differences in types of IGR variants among them. However, the characteristics of Korean isolates such as the IGR II variant between the I73R and I329L genes, and CVR 1 of the B602L gene, have been detected in European countries and China—Pol14/Sz, China SY-18, China/Pig/HLJ/2018, and China/2018/AnhuiXCGQ. Recently, a CVR 1 variant with single nucleotide polymorphism (SNP) 1 strain was identified in Estonia, and CVR 1/SNP 2 and CVR 1/SNP 3 are circulating in Poland and Lithuania [15]. This CVR 1 profile of genotype II was also reported in Africa [16,17], where there are various genotypes and CVR types, with different CVR types (variant tetrameric amino acid repeats) within the same genotype [6,18,19].

**Figure 4:**
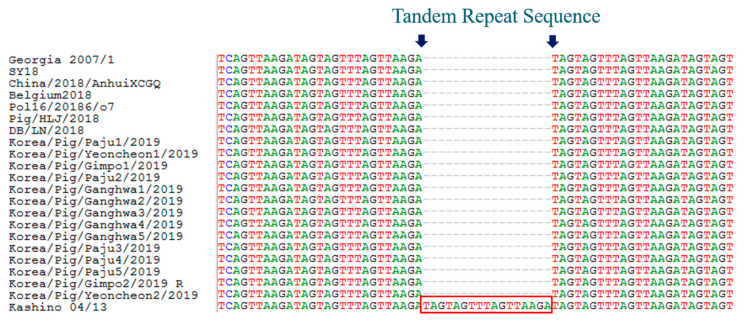
Intergenic region (IGR) of MGF 505 9R/10R analysis of ASFV isolated in domestic pigs of South Korea, 2019.

The TRS insertion in the IGR of MGF 505 9R/10R is a new marker of genetic variation among ASFV of genotype II [5]. There are two types according to the insertion: MGF-1 type without insertion and MGF-2 type with a new long insertion of 17 nucleotides (GATAGTAGTTCAGTTAA). MGF-2 type has been observed in seven Russian isolates from Vladmir and Tver regions, and in nine Polish isolates [20]. For the analysis of Korean isolates, we used a set of primers that amplified a ~500 bp fragment located in the IGR of MGF 505 9R/10R. The result showed that all Korean isolates have the characteristics of MGF-1 without insertion in this region, and the sequence of IGR of MGF 505 9R/10R was 100% identical to that of Pol16 20186 o7, Belgium 2018/1, Pig/HLJ/2018, DB/LN/2018, and China/2018/AnhuiXCGQ (Figure 4, Table 2). Recently, the MGF-3 type, which contains two identical insertions in MGF 505 9R/10R, was found in the Tver region of Russia (Tver1112/Zavi) [20].

Since the first outbreak of ASF in South Korea, we isolated 14 ASF viruses from infected domestic pig farms and all the isolates showed the ability of HAD. Through the molecular characterization, all 14 Korean isolates showed the same characteristics of genotype II-IGR II variant-CVR 1, MGF-1, and serogroup 8. These results suggest that only one variant of the ASF virus caused the outbreaks of ASF in the 14 pig farms in South Korea. Moreover, the results suggest that the molecular characteristics of Korean isolates are similar to some European and Chinese isolates.

The genome of ASFV is much larger and more stable than that of other viruses. Due to these characteristics, the genotyping of ASFV has been done based on the highly conserved B646L gene. However, there are very few genetic variabilities in the B646L gene among the same genotype; other genetic regions have been investigated for the intra-genotype differentiation, such as the CVR of the B602L gene as a less-conserved region [21]. Although we compared the CVR of 14 isolates in this study, the geographical and temporal distribution of the ASF outbreak in South Korea is not large. Therefore, it is necessary to compare a greater number of ASFV isolates in further study. Furthermore, the whole-genome sequence using next-generation sequencing (NGS) was developed and revealed new genetic markers such as the IGR of MGF 505 9R/10R [5]. In comparison to other important animal disease viruses such as foot and mouse disease virus (FMDV) and highly pathogenic avian influenza virus (HPAIV), it is very difficult for ASFV to have the source of introduction and route of spread identified by the genetic analysis, due to its genetic stability. Nonetheless, as numerous studies have confirmed several genetic markers of ASFV, we could get more genetic information with the help of whole-genome sequencing.

For Korean isolates of ASFV from pig farms, in this study we confirmed that all 14 isolates have the same nucleotide sequences on 5 marker regions of ASFV genotype II; however, further study will be needed to investigate any differences in detail between isolates with whole-genome sequencing using NGS.

## Figures and Tables

**Table 1:** Summary of the isolation and genetic characterization results from ASFV isolates 2019 in South Korea.

	Isolate Name	Isolate Organ	PCR *C*_t_ Value	HAD	P72 Genotype	IGR_I73R-I329L_	CVR Type	IGR_MGF505 9R10R_
1	Korea/Pig/Paju1/2019	Spleen	17.1	Positive	II	IGR-II	CVR 1	MGF-1
2	Korea/Pig/Yeoncheon1/2019	Spleen	17.2	Positive	II	IGR-II	CVR 1	MGF-1
3	Korea/Pig/Gimpo1/2019	Blood	15.4	Positive	II	IGR-II	CVR 1	MGF-1
4	Korea/Pig/Paju2/2019	Spleen	15.3	Positive	II	IGR-II	CVR 1	MGF-1
5	Korea/Pig/Ganghwa1/2019	Blood	13.3	Positive	II	IGR-II	CVR 1	MGF-1
6	Korea/Pig/Ganghwa2/2019	Blood	15.4	Positive	II	IGR-II	CVR 1	MGF-1
7	Korea/Pig/Ganghwa3/2019	Blood	15.5	Positive	II	IGR-II	CVR 1	MGF-1
8	Korea/Pig/Ganghwa4/2019	Blood	16.0	Positive	II	IGR-II	CVR 1	MGF-1
9	Korea/Pig/Ganghwa5/2019	Spleen	17.6	Positive	II	IGR-II	CVR 1	MGF-1
10	Korea/Pig/Paju3/2019	Spleen	18.1	Positive	II	IGR-II	CVR 1	MGF-1
11	Korea/Pig/Paju4/2019	Blood	15.4	Positive	II	IGR-II	CVR 1	MGF-1
12	Korea/Pig/Paju5/2019	Spleen	16.4	Positive	II	IGR-II	CVR 1	MGF-1
13	Korea/Pig/Gimpo2/2019	Spleen	18.1	Positive	II	IGR-II	CVR 1	MGF-1
14	Korea/Pig/Yeoncheon2/2019	Blood	15.5	Positive	II	IGR-II	CVR 1	MGF-1

**Table 2:** Comparison of molecular characterization between ASFV genotype II.

Name	P72 Genotype	IGR_I73R-I329L_	CVR Type	IGR_MGF505 9R-10R_	GenBank No.	Ref
Korea/Pig/Paju1/2019	II	IGR II	CVR 1	MGF-1	MT748042.1	
Georgia 2007/1	II	IGR I	CVR 1	MGF-1	FR682468.1	
China SY-18	II	IGR II	CVR 1	MGF-1	MH766894.1	
China/2018/AnhuiXCGQ	II	IGR II	CVR 1	MGF-1	MK128995.1	
Pig/HLJ/2018	II	IGR II	CVR 1	MGF-1	MK333180.1	
DB/LN/2018	II	IGR II	CVR 1	MGF-1	MK333181.1	
China/Jilin/2018/boar	II	IGR I	CVR 1	NA *	MK189457.1, MK214681.1	[12]
China/Guangxi/2019	II	IGR III	NA	NA	MK670729.1	[13]
Belgium2018/1	II	IGR II	CVR 1	MGF-1	LR536725.1	
Pol16_20186_o7	II	IGR II	CVR 1	MGF-1	MG939583.1	
Russia/Kashino;04/13	II	IGR I	CVR 1	MGF-2	KJ747406.1	[5]

* NA: not applicable.
